# Cholera

**Published:** 1849-04

**Authors:** 


### CHOLERA.

The steamboats ascending the Mississippi and its tributaries are infested with cholera. Upon nearly all that have arrived at Louisville from New Orleans during the last fortnight, cases, some of them fatal, are reported to have occurred. The disease is prevalent at New Orleans, but not to an extent to excite any alarm. The Nashville papers of the 24th March mention several cases of the epidemic as having appeared in that city, but only one death is reported. During the week ending the 19th of March, twenty-six deaths from cholera took place in St. Louis. The epidemic has made its appearance at the Jefferson Barracks, and from the 13th to the 19th of March, twenty-seven deaths from the disease occurred among the troops stationed there. A few cases have originated in Louisville, and some deaths have been attributed to the disease within a few days; but thus far it has not manifested an epidemic tendency unless the frequent occurrence of bowel complaints may be regarded as such. These affections, in some instances, have been severe, but a prompt resort to remedies has quickly subdued them.

The March number of the New Orleans Medical and Surgical Journal states, in reference to the decline of cholera, that the deaths for the week ending the 17th of February were 64, while for the previous week they were 80, showing a diminution of 16 deaths weekly. The pestilence was not subsiding so rapidly as it did in 1832.

The editor mentions a disease which has appeared in some parts of Louisiana, near sluggish streams and bayous, wearing the type of pneumonia, and which is reported to kill in a few hours, the patient becoming prostrate, powerless, and cold, from the first hour of the attack. He suggests that this may be cholera in a masked form. No vomiting or purging attends it.

Cholera as it recedes from the river is found to assume a less virulent character.

